# Spaces for women: Rethinking behavior change communication in the context of women's groups and nutrition-sensitive agriculture

**DOI:** 10.1016/j.socscimed.2021.114282

**Published:** 2021-09

**Authors:** Carly E. Nichols

**Affiliations:** University of Iowa, 312 Jessup, Hall Iowa City, IA, 52245, United States

**Keywords:** Self-help groups, Participatory learning and action, Maternal and child health, Women's health, Health promotion ethics

## Abstract

Recently, nutrition-sensitive agriculture programs have taken aim at malnutrition's multi-sectoral roots through re-diversifying agricultural production while integrating women's empowerment and nutrition behavior-change communication components. For these integrated nutrition-sensitive agricultural programs, women-led self-help groups have emerged as promising platforms for program delivery. Yet, while well-designed nutrition behavior-change communication has been successfully used in self-help groups, and is central to nutrition-sensitive agriculture, it can take many forms. These vary widely in their theoretical and ethical underpinnings, communication strategies, and theory of change. As nutrition-sensitive agriculture continues to proliferate, it is critical to better understand how women interact with different behavior-change messages and how to engage individuals in ethical, effective ways. This paper analyzes qualitative data collected from a nutrition-sensitive agricultural project in India that used participatory storytelling to generate knowledge and awareness about malnutrition among women. Drawing from data across two sites, the paper analyzes why certain messages generated more discussion among women then others. We find self-help group women were drawn to topics of early marriage and diet diversity because they emotionally connected to them, and felt they were relevant to their lives with high perceived pay-off and actionability. While other topics on gender and health also provoked emotional, lively discussions, the stories were less effective due to their complexity, which were difficult for volunteer facilitators to communicate. We conclude that there is unmet demand among women in rural India for structured spaces to discuss gendered aspects of health and diet, and nutrition-sensitive agricultural programs could benefit from focusing attention here.

## Highlights

• Engages debates on health behavior change communication (BCC) ethics and efficacy.
• Examines modified participatory learning and action (PLA) run in women's groups.
• Finds women were drawn to simple, actionable messages they emotionally related to.
• Insufficient investment in volunteers' PLA facilitation skills hampered impact.
• There is need for more BCC focused on women as women, rather than mothers.

## 1 Introduction

While there has been progress in improving nutrition over the past two decades, this has been slow and unevenly distributed. This is especially true in India, where economic growth has not resulted in commensurate improvements in nutritional indicators, particularly among the most marginalized communities. Resultantly, nutrition-sensitive agriculture has emerged as a paradigm to address malnutrition's multi-sectoral roots in the context of this country's growing economy (Ruel and Alderman, 2013; Ruel et al., 2018). Nutrition-sensitive agriculture is a “food-based” approach to agricultural development that emphasizes the multiple social, economic, and health benefits from consuming a diversity of nutrient-rich foods, and is specifically designed with nutritional goals in mind (FAO, 2014; Ruel and Alderman, 2013). Although there are many types of nutrition-sensitive agriculture programs, a recent review finds the most successful integrate women's empowerment and behavior-change communication components alongside attempts to re-diversify agricultural production to include more nutrient-rich foods and livestock (Ruel et al., 2018). Among nutrition-sensitive agricultural programs with women's empowerment goals, the expansive networks of women-led self-help groups across South Asia have emerged as promising platforms for program delivery (Malapit, 2019; Ruel et al., 2018: 148). Researchers suggest that “layering” nutrition behavior change communication onto self-help groups' existing agricultural and income activities may be a cost-effective way to address nutrition through multiple pathways while also reaching difficult to access populations (Gugerty et al., 2018; Kumar et al., 2018). While behavior change communication is seen as critical to achieving nutritional gains within nutrition-sensitive agriculture, it remains difficult to create sustained changes in habitual behaviors shaped by not only knowledge, beliefs, and self-efficacy, but also structural poverty and social norms (Aboud and Singla, 2012; Ruel et al., 2018). As such, behavior change communication takes many different forms, differing widely in terms of theoretical and ideological underpinnings, communication strategies, delivery methods, as well as the practices that are targeted. Thus, large debates remain surrounding how to ethically and sustainably use behavior-change communication to achieve improvements in health and nutrition practices and outcomes.

Much research has examined behavior-change communication programs' impact on women's knowledge, beliefs, and practices, yet there has been decidedly less work that examines how and why health messages resonate with women from their own perspectives (Lentz et al., 2019). As global development focuses more squarely on achieving equity, there has been greater focus on the power dynamics inherent in health interventions and how to achieve impact in ethically sound ways (Lentz et al., 2019, McPhail-Bell K. Bond C. Brough M. & Fredericks B., 2015). As a more radical offshoot of behavior-change communication within India, a model of participatory learning and action has achieved much success among indigenous belt of eastern India over the past decade. Derived from Paulo Freire, participatory learning and action uses dialogical learning methods to enable marginalized communities to identify, analyze, and address their (health) concerns and the power relations they are embedded within (Prost and Colbourn, 2013). Compared to more traditional didactic behavior change methods, participatory learning and action is seen as an empowering (and perhaps a more ethical) approach to behavior change among historically oppressed populations because it may avoid reproducing the paternalistic power relations that created harm within indigenous communities or others subjected to colonial rule (McPhail-Bell K. Bond C. Brough M. & Fredericks B., 2015, Prost and Colbourn, 2013).

In contrast to the core Freirean approach, the most widespread participatory learning and action interventions within self-help groups have followed a highly structured approach using pre-defined objectives (e.g., reducing neonatal mortality) and paid facilitators to deliver participatory modules in groups formed with new and expecting mothers. Highly-structured participatory learning and action, therefore, may not philosophically align with organizational commitments to more radical grassroots participation, where communities have greater power in defining project goals. Moreover, questions have also risen about whether the use of paid, trained facilitators detracts from program 'sustainability' by creating dependence on paid actors rather than embedded agents of change (Gugerty et al., 2018). As a result of these concerns, there has been substantial interest as to how a less-structured and volunteer-facilitated participatory learning and action approach might work within the large networks of already-existing self-help groups in India (Sethi et al., 2017; Gugerty et al., 2018; Gupta et al., 2019).

This paper works to fill these gaps in knowledge through analyzing qualitative data collected from a nutrition-sensitive agriculture project run through self-help groups in marginalized, Indigenous-dominant districts of East India that are using a participatory approach to health and nutrition behavior change communication. It addresses two key questions:

(1) Can volunteer-facilitated participatory behavior change communication based in the principles of participatory learning and action work within pre-existing self-help groups?
(2) How can behavior-change messages be constructed to best resonate in the context of women-focused nutrition-sensitive agriculture programs?

In addressing these questions, the aim is to not just understand what approaches are *effective*, but to understand why certain messages resonate more than others, and whether they are empowering and respect participants' cultural traditions, and local knowledge systems (Tengland, 2012, Dutta, 2007). The paper proceeds in five parts. Section two reviews the literature on behavior-change within nutrition-sensitive agriculture and self-help group-led interventions, respectively, and brings them into conversation with more critical studies of health promotion centered in behavior-change programs inspired by participatory learning approaches. Section three details the program intervention, study sites, and methodology, and is followed by the empirical findings. Section four discusses the results, which find that while self-help group members emotionally connected to gender- and food-related modules, and sometimes had robust dialogical analysis, volunteers’ uneven facilitation skills resulted in a shift towards easier-to-communicate didactic messaging that caused the project to fall short of achieving the more radical aims of participatory learning and action approaches. Section five examines how behavior change communication might more effectively work in the context of nutrition-sensitive agricultural projects focused on women.

## 2 Literature review

There are growing literatures on both nutrition-sensitive agricultural programs (Ruel and Alderman, 2013; Ruel et al., 2018) and the potential to use women's self-help groups as platforms for a range of development programs, including health promotion activities (Brody et al., 2017; Gugerty et al., 2018; Kumar et al., 2018). These literatures both focus on women-centered interventions and improving health and nutrition, yet their evidence bases have not been in direct conversation.

However, while nutrition-sensitive agricultural projects tend to utilize behavior change communication focused on broadcasting simple messages through multiple communication channels (Ruel et al., 2018), projects conducted with self-help groups have more often used the participatory learning and action approaches, described above (Kumar et al., 2018). The stream of behavior change communication that nutrition-sensitive agricultural projects have more often used is underpinned by behavioral theories of change that seek to transform individuals’ knowledges, beliefs, and capacities to act, while addressing potential barriers at levels of individual, family, community, health system, and environment (USAID, 2015). These programs emphasize selecting a few key behaviors that are most pressing in the community, and then broadcasting simple messages to audiences (including men and mothers-in-law) through multiple channels such as interpersonal communication, health workers, and media. The focus is on “small do-able” actions, and health workers are trained in “negotiating for behavior change” approaches to counseling (Nordhagen et al., 2019; Olney et al., 2016, USAID, 2015).

While participatory learning and action approaches also understand social and behavior change as non-linear and embedded within larger ecological, socio-cultural, and economic constraints, they move away from didactic communication persuading women to change, towards an empowerment approach that gives women space, information, and tools to identify, analyze, address, and assess health concerns (Prost and Colbourn, 2013). Narratives and storytelling are used to help participants draw connections between socio-economic oppression and health status, with the goal of mobilizing communities to shift power structures and relations (Rath and Nair, 2010). Storytelling groups may also provide therapeutic spaces for processing, affirming, and validating one's experience while also educating others. (Banks-Wallace, 1998). Moreover, relatable narratives may be cognitively easier to process than directly persuasive fact-based appeals, giving storytelling both emancipatory and strategic relevance (Hinyard and Kreuter, 2007; Larkey and Hecht, 2010). In theory, participatory learning and action along with narrative-based communication avoids individualizing health outcomes as personal failings or “ignorance” and widens the circle of responsibility to include historical and contemporary marginalization (Tengland, 2012).

Yet, while participatory learning and action approaches are community-driven with each group identifying the actions they want to take, the overall goals are pre-determined, such as reducing neonatal mortality (Prost and Colbourn, 2013) or improving child growth (Nair et al., 2017). Participatory learning projects' highly structured 16–20 meeting format delivered by paid, well-trained facilitators has led Gugerty et al. (2018) to hypothesize that success may be due to the use of trained facilitators following structured protocols, and also question whether behavior changes will sustain once the program ceases. Despite questions about participatory learning and action-inspired programs’ structured approach and pre-determined goals, it remains markedly different from the behavior change approaches used in documented nutrition-sensitive agriculture projects. While both message-oriented behavior change communication *and* participatory learning and action approaches have amassed impressive evidence bases in the last decade (Sanghvi et al., 2017, Prost and Colbourn, 2013), they have different ethical and political commitments that are important to consider when engaging marginalized populations.

Tengland (2012) usefully traces the ethical implications of both traditional behavior change communication and empowerment approaches. He argues that while traditional behavior change approaches communicate risk to persuade people to change on the grounds of harm reduction, their paternalism risks violating individuals’ autonomy to make educated choices for themselves. More troublingly, they may introduce power dynamics that belittle information recipients as ignorant or “lesser than.” The “prevention paradox” (Rose, 2001) may also occur where marginalized communities gain knowledge about optimal health behaviors yet do not have the economic or social means to enact them.

These arguments become more pointed when promotion is carried out among marginalized communities exposed to intersectional forms of oppression. Here, scholars argue that paternalistic health promotion delivered didactically to Indigenous communities may recreate problematic colonial power dynamics and have little resonance within communities that remain wary of outsider intrusion (Dutta, 2007, McPhail-Bell K. Bond C. Brough M. & Fredericks B., 2015). Literature examining women-focused health interventions provides a similar set of cautionary findings. Warin and Zivkovic (2015) examines how temporal orientations towards the future in preventative health promotion often do not resonate with poor women who remain concerned with the “here and now”. She questions how unactionable behavior change communication messages around child feeding may do more harm than good by further stigmatizing already marginalized women.

Notably, behavior-change programs in both nutrition-sensitive agriculture and self-help group-led programs have focused women primarily as mothers and caretakers (Malapit, 2019; Ruel et al., 2018). This is surprising as nutrition-sensitive agriculture's conceptual frameworks acknowledge women's integral role in agriculture, and that their ability to control income, assets, and their time-use is crucial to ensuring the health of *themselves*, and their children (Ruel et al., 2018). Malapit (2019) argues nutrition sensitive agricultural programs' behavior-change focus on child feeding is a missed opportunity to address gendered health disparities, more broadly, as an important pathway between agriculture and nutrition. Ruel et al. (2018) also encourage nutrition-sensitive agricultural programs to rethink behavior change communication so that it is more relevant to women, does not create excessive time burdens, and perhaps shifts away from a narrow focus on infant and young child feeding. Similarly, feminist scholars have long contended early self-help groups' radical feminist goals of collectively transforming power structures have often been reduced to a narrow focus of producing responsible financial subjects or dutiful mothers (Batiwala, 2007). To be sure, these critiques are not unique to self-help groups or nutrition-sensitive agricultural programs, as they have been a frequent shortcoming of women-focused development, more broadly (Wilson, 2015). Lentz et al. (2019) echo these contentions to argue that within the nutrition-sensitive agriculture paradigm it is necessary to listen to and address women's own concerns *as women* (rather than mothers) from a human's rights perspective and as strategic policy, because women are experts on their own health situations.

Thus, from an ethical stance, Tengland's “empowerment” approach - where communities are given control over problem definition, solutions, and actions while professionals act as enablers/helpers -fares better than traditional behavior change communication, though it also has drawbacks. Most pressing, the focus on consensus building is time-consuming, which may increase project budgets or be problematic if more urgent action is needed. While individual autonomy is respected, limited control over processes may lead groups to pursue strategies the professional believes are misguided. Finally, as the broader literature on participatory development has long surmised, Tengland contends that perfectly egalitarian spaces are difficult to achieve since powerful voices within the group may always retain some control and thus direct discussion.

These insights on the potential and pitfalls within standard behavior change communication and participatory approaches are critical to more wholly understanding the trade-offs of behavior change strategies in self-help groups. The structured approach of participatory learning and action programs is relevant to examine in this light, as its pre-determined goals and use of a paid facilitator might undermine the empowerment approach's more radical goals. A structured approach may address empowerment's potential shortcomings of being time-consuming, dominated by powerful voices, or derailing into misconceived action. Yet, for organizations committed to community-driven development, a less structured participatory approach may be compelling. Less structured approaches, such as the one investigated in this study, also give opportunities to better listen to women's own perspectives and examine what issues resonate with them. Such insights may be used to examine how health behavior change communication might be more effective and equitable within the marginalized communities it serves.

## 3 Intervention description

This intervention was implemented by the non-governmental organization Professional Assistance for Development Action (PRADAN) and the Public Health Resource Society (PHRS). PRADAN has extensive networks of livelihood-oriented SHGs across seven states of India. In 2013 they partnered with PHRS to address malnutrition across eight project sites through agricultural diversification programs and nutrition behavior-change communication. The goal was to enable women to identify and analyze the underlying causes and practices associated with malnutrition, and to trigger a set of actions to reduce malnutrition at the level of self, community, and health system.

The behavior change communication curriculum was delivered through set of iteratively developed perspective-building ‘micromodules’ (see Table 1).

**Table 1:** Description of intervention curriculum.

Module Number and Story Name	Topic	Module Summary	Key Messages
1. Soni's Story	Early Marriage	Soni is married early by her parents and gets pregnant at young age. She works throughout her pregnancy without taking healthy food or rest and gives birth to malnourished child, Madhu.	-Do not marry daughters until 18 years of age

2. Madhu's Story	Maternal Health	Soni insists Madhu completes her education. She marries her off after 18 years and ensures she completes recommended practices for a healthy pregnancy. Madhu's child is healthy and tall.	-Register birth with health-worker *-* Seek prenatal care

3. Our Foods	Dietary diversity	Participants play a food sorting game to learn starches give energy, proteins build muscle, and vegetables/fruits provide protection from disease.	-Eat vegetable, protein, and starch together 1x/day - Eat meals 3x per day

4. Silvanti's Dream	Kitchen gardens	Silvanti wants to eat tri-color meals but is poor and does not have access to market-bought vegetables. She instead cultivates them on her homestead.	-Grow variety of nutritious vegetables on homestead

5. Ropni's Story	Women's Health, Wellbeing, and Disease	Ropni is verbally abused by her husband. She subsequently feels mental tension, doesn't eat properly, and falls ills. She goes to the doctor, where she is diagnosed with anemia and prescribed iron tablets. Afterwards Ropni contracts malaria due to her weakened immune system, and learns about malaria prevention.	-Verbal abuse can cause health problems - Take rest midday; do not overwork - See a doctor if ill -Take iron tablets & eat greens if anemic -Use a mosquito net and keep area around home clean

6. Maya's Story	Family Planning & Reproductive Health	Maya has no birth plan and has a frightening, life-threatening birthing experience. She subsequently learns about birth preparedness, contraceptives, and the benefits of small family size from the health worker.	-Use contraceptive of choice to avoid unwanted births -Have a birth readiness plan -Smaller family sizes have many benefits

7. Sushila's Story	Breastfeeding and Neonatal care	Sushila learns not to wash her newborn child, and to keep her wrapped in a clean cloth. She feeds her baby colostrum and then practices exclusive breastfeeding until the baby is 6 months. She troubleshoots breastfeeding problems.	-Do not wash newborn babies, but wrap in towel -Give newborn colostrum and continue exclusive breastfeeding for 6 months

8: Urmila's Story	Complementary Feeding and Childhood Disease	Urmila has to do agriculture work and her baby's growth falters due to lack of complementary foods and diarrhea. Urmila learns about oral rehydrating salts (ORS) for diarrhea and the type/frequency of complementary food necessary for healthy child growth. She learns to keep her child away from cookstove smoke to prevent pneumonia.	-After 6 months, feed children nutrient rich foods frequently -Use ORS for diarrhea -Keep away from cookstove smoke to prevent pneumonia

PRADAN/PHRS staff and self-help group leaders initially conducted community needs assessments to inform curriculum content, which was piloted in several sites in 2013–15. Program designers observed didactic simple messaging neither resonated with women nor was effective in propelling behavior change. While PRADAN was aware of the participatory learning and action model's success, they preferred to give their self-help groups more autonomy to focus on issues relevant to them, rather than provide a pre-defined outcome for them to address (e.g. neonatal morbidity). They, thus, offered up a wide-range of modules to build knowledge and capacity around far-reaching health issues, which communities could address per their relevance. The micromodules consisted of engaging stories and a shortened participatory learning and action cycle conducted over two meetings. Each story featured a woman who faced health- or nutrition-related challenges and then learned recommended health behaviors, faced barriers to change, and ultimately succeeded in changing her practices. After stories were communicated, women were asked to share their experiences relating to the story. Facilitators posed specific questions encouraging situational analysis so that women could analyze the issue and overcome bottlenecks to action. Meetings ended with women pledging to take certain recommended action items. In subsequent meetings, women were supposed to discuss the successes and barriers they faced in implementing the action pledges, thereby completing the participatory learning and action cycle.

The first four micromodules were introduced in fall 2016 through three-day training programs for PRADAN/PHRS professionals and a team of local nutrition mentors who were hired through a competitive process in each site to ensure smooth implementation. Mentors and PRADAN/PHRS professionals then helped self-help groups select members as nutrition volunteers. These women received three-day residential trainings on micromodules, which were accompanied by follow-up refresher trainings. With mentor support, nutrition volunteers were envisioned to facilitate micromodules among the self-help groups in their village hamlets, and also emerge as community resource persons knowledgeable about health. This stands in contrast to the more structured participatory learning and action programs where facilitators are paid monthly wages or per training incentives (e.g. see Gope et al., 2019; Rath and Nair, 2010).

Micromodules five through nine were developed in response to feedback from mentors and nutrition volunteers about issues they felt were locally important. There were requests to address many issues and latter micromodules featured longer stories with wider ranges of messages. All nutrition actors were trained on the second set of micromodules in three-day trainings in fall 2017. Finally, in fall 2018 three “review modules” that consolidated earlier learnings were introduced. However, these had not been rolled out in all study sites prior to this study's data collection, which took place in May–June 2019.

## 4 Study sites and methodology

This study focused on two of eight PRADAN nutrition sites: Bastar District, Chhattisgarh and Purulia District, West Bengal^1^. These sites were purposefully chosen to have maximum variability as program evaluation data and PRADAN's institutional history differed between sites.

Purulia is one of PRADAN's oldest, most established sites (18-year engagement), whereas Bastar is relatively new (8-year engagement). Purulia and Bastar's socioeconomic demographics are markedly different. Purulia's population is predominantly low-caste Hindus, whereas Bastar is heavily dominated by Gond and Halbi Indigenous groups, also known as Scheduled Tribes (see Table 2). The aim in selecting these sites was to understand common and divergent enabling factors in barriers of the same program in different environmental and socioeconomic contexts.

**Table 2:** Block level census statistics (2011 census of India).

	Purulia District Block-level data		Bastar District Block-level data	
	Total	Female	Total	Female
Population	137,143	67,048	79,360	40,389
Literacy Rate	66.2%	43.8%	38.3%	24.8%
Scheduled Caste	12.4%	12.3%	0.3%	0.3%
Scheduled Tribe	11.4%	11.4%	82.9%	83.2%

The same protocols for sampling and data collection were used across sites. We interviewed the block-level staff responsible for the nutrition intervention, including PRADAN professionals and mentors. All respondents were female except for the Purulia mentors, who were all male, and three PRADAN professionals (1 in Purulia, 2 in Bastar). Except for one unmarried Bastar mentor, all mentors were married, and their households were connected to self-help groups. They had all completed secondary education and were highly literate.

We selected 12 nutrition volunteers to interview and an additional 8–10 for focus groups based on PRADAN's monitoring data and conversations with block staff (see Table 3).

**Table 3:** Interviews and focus group disussions conducted in study sites.

	PRADAN Block Professionals	Mentors	Nutrition Volunteers	Self-help Group Members	Total
Interviews					
Purulia	2	5	12	14	33
Bastar	3	4	11	13	31
Focus Groups (5–6 participants/group)					
Purulia	–	1	2	–	3
Bastar	–	1	2	–	3

Volunteers were sampled to achieve maximum variation based on proximity to the block town, group longevity, and the volunteers' performance assessments. PRADAN staff graded the volunteers' performance, and we selected six women from the low-medium range and six graded high. All volunteers, except one illiterate Bastar woman, had completed primary education and were at least semi-literate. Volunteers were almost all married with children, although we interviewed three unmarried Bastar volunteers.^2^ In six volunteers’ villages, we purposefully selected 2–3 women from different self-help groups for interviews, trying to get variation in terms of age, education, and relative wealth (see Table 4).

**Table 4:** SHG sample characteristics.

	Jhalda (n = 14)	Bastar (n = 13)
Average Age of Respondent	33 (min: 19, max: 45)	28 (min: 21, max: 39)
Average Household Size	4.5	5.5
Average # of children	2.5	2.7
# of respondents in combined family	6	6

Data was collected through semi-structured interviews and focus groups. Interview guides were written in English and translated by native speakers of the local language. They were piloted in Purulia and adjusted accordingly. Interviews consisted of open-ended questions asking self-help group members, nutrition volunteers and mentors about their experiences either telling or listening to stories. We asked how hearing the stories made them feel and probed around whether they or others had begun to implement practices. For mentors and volunteers, we asked how they personally felt about the stories, and how they were received in different self-help groups. A research assistant for Purulia and Bastar transcribed and translated interview and focus group audio. The transcriptions were uploaded to MAXQDA 2018 software for analysis using inductive coding. Data was read and notes were taken on emerging themes. Particular attention was paid to the different ways in which women discussed their experiences with stories. While women sometimes recalled messages and stories in a rote manner, other times they were excited to discuss their experience with the material. We interpreted this latter type of response as evidence that women more fully internalized messages and drawn connections to their lives. After an initial coding, sets of commonly coded segments were retrieved and further analyzed to describe variability. The analysis consists of summarizing the dominant themes.

This study has several limitations. Different research teams were used between sites, and the Purulia interviewer was more experienced than the Bastar interviewer. There were language barriers in Bastar, as the interviewer did not speak Gondi, which many women in more interior areas speak, so we only interviewed women who spoke Halbi or Hindi. Lastly, while there is always possibility of social desirability bias given that the interviews centered on normative health behaviors, the research team felt respondents were largely forthcoming. The data are not an exhaustive survey of the respondents’ knowledge, but instead capture the messages that resonated most strongly.

## 5 Findings

The findings are structured in two parts. Part one examines how emotionally-relatable stories created opportunities for self-help group members to explore the social determinants of poor health, but demonstrates not all members had opportunities to engage due to nutrition volunteers' uneven skills in telling stories and guiding dialogical learning. The second section examines why early marriage and dietary diversity modules had higher traction among women despite variability in nutrition volunteers’ facilitation skills.

### 5.1 Relatability and relevance: transportation and the importance of skilled facilitation

We found that being able to emotionally relate to a story was critical to spurring further analysis and discussion in self-help groups. Across sites, mentors concurred gender-focused stories (e.g. early marriage, women's health, reproductive health) were most ‘popular’ because they addressed issues relevant to many women's lives. For example, a Purulia mentor stated:

*I think they understand those stories best which they can relate to their own lives. For example … when we discuss [Maya's] story they relate to their own lives and understand that [having more than 2 children] is the reason for their declining health.* (Purulia mentor, male, age 28, 6/3/2019d)

This mentor's insight that “relatable” stories were ones that women had personal experience with was evidenced further in the way women responded to questions about the curriculum. We found that when self-help group members discussed the more gender-focused stories, they did not just personally identify, but also spoke more fluently and with greater interest than when discussing more ‘technical’ health information (e.g. optimal infant and child feeding). Thus, a central point is that “relatable” stories did not just have realistic, culturally-relevant plotlines, but also a particularly strong emotional valence. For example, in Bastar, several women spoke about how Ropni's story about women's health and wellbeing provoked much discussion among women. This nutrition volunteer's comment was broadly representative:

*SHG members shared their own life experiences while thinking of Ropni's story. Ropni used to do everything, she used to think a lot and have lots of tension and hurt. And her daughter was facing difficulties as well. We all said, we won't do like that!! Despite everything, the husband wasn't understanding his wife. We think a lot about it. We think we will never do like that.* (Bastar volunteer, female, age 22, 5/31/2019a).

Many women also spoke emotionally about early marriage, such as this Purulia respondent:

When we heard the early marriage story and the subsequent problems, we felt bad. We thought about ourselves and we thought this should not happen to our girls. They should not get married before turning 18. We [married early] and we made mistakes. (Purulia SHG member, age 45, 6/10/2019).

Both of these quotes illustrate how both stories about women's wellbeing and early marriage were not merely “relatable”, but provoked particularly *emotional* responses that were not observed in responses about child feeding stories. Older women (like in the second quote) connected to these stories due to their own life histories, which were sometimes traumatic, while younger women were moved to vow to do things differently (such as in the first quote). In articulating cause and effect linkages, stories opened up space for women to draw connections between earlier hardships and present health experiences. Many women expressed collective grief, and subsequent motivation to make change for future generations. Although women drew connections between poverty or patriarchy and health, there were few discussions that connected health to larger political economic structures. Some women also expressed shame around their “ignorance” rather than seeing outcomes as broader failings of the state or society, as participatory learning and action programs intend.

Given the complexity of facilitating discussions in empowering, non-judgmental ways, we found gender-focused modules were more effectively facilitated by experienced trainers – usually PRADAN or PHRS professionals. While mentors and some nutrition volunteers could also lead modules effectively, the majority had insufficient soft-skills training to confidently facilitate. PRADAN/PHRS professionals reported that stoking an emotional connection meant that volunteers and mentors had to remember the stories in full and tell them with “appropriate feeling” (Purulia staff, 6/11/2019). Purulia mentors observed that stories were less effective when nutrition volunteers forgot and had to read word for word, rather than recite from memory in an engaging way. Latter micromodules' longer stories – such as Ropni (women's health) and Maya (family planning and safe delivery) – were especially challenging for volunteers to remember. Many group members and nutrition volunteers became confused when recalling these stories, and volunteers reported they often resorted to didactically disseminating the messages. While this approach was a logical solution, it perhaps influenced whether women could relate to the messages.

Indeed, while personal or emotional identification with story characters is central to effective narrative-based communication, “transportation” – or the quality of becoming completely immersed – is the second key mediating mechanism (Larkey and Hecht, 2010). This is especially true for individuals with low bandwidth for complex cognitive processing, which was often the case for self-help group members, who would be preoccupied with childcare or pending household and agricultural work. Bastar mentors were clear that it was difficult for women to relate to didactic messages. One mentor said, *“some self-help group women only listen with their ears, not their hearts”* (Bastar mentor, age 28, 6/12019), while another reported *“some self-help group women think: ‘it is your words and our ears’ and they don't pay attention”* (Bastar mentor, age 32, 5/27/2019a). Thus, while many of the modules may have promoted “identification,” perhaps only the shorter, easier to communicate stories allowed for “transportation.”

Purulia PRADAN professionals estimated that approximately 40% of volunteers had some sort of mastery over stories (Purulia staff, 6/72019). In Bastar, where illiteracy rates were higher and there was less training, there were few volunteers who could engagingly tell stories (interview, 5/29/2019). One volunteer illustrated: “*While telling stories, we also forget things. The whole thing is difficult to remember …. I forget half of it”* (Bastar volunteer, age 29, 5/31/2019b). While all volunteers in Purulia were literate, many struggled in telling stories because too much time had elapsed. As one stated, *“we have so many household works, it is hard to remember”* (Purulia volunteer, age 33, 6/102019).

Interestingly, nutrition actors who drew from personal experience were more effective facilitators than those who did not. This may have lessened stigma around past “mistakes” and created open spaces for sharing. When group members could identify with the *storyteller* in addition to story characters, it may have facilitated deeper learning through intensifying the experience of identification (c.f. Larkey and Hecht, 2010). In one example, a Bastar volunteer spoke frankly about how having a young, underweight child motivated her to help other women so their children do not share the same fate (FGD 6/1/2019). Several Bastar mentors expressed similar mixes of emotions when they saw commonalities between stories and their own lives.

While gender-focused modules touched a deep need for women to process life events, respondents recalled the dietary diversity module with the highest frequency and most detail. As the principal food preparers and farmers, women's lives are centered around food and agriculture. Women had expertise on the subject matter, yet had not developed vocabulary to describe the functional significance of different foods until they examined benefits from starches, proteins, and vegetables/fruits in the micromodule. Compared to other messages (e.g. infant and child feeding or maternal health), dietary diversity was relevant for everyone. The majority of respondents were also enthusiastic about planting kitchen gardens, which consisted of 8–10 different kinds of vegetables cultivated organically.

Notably, while there were structured spaces for women to discuss infant and child feeding and maternal health – such as the village childcare center and with village health workers - there were few spaces where women could speak about *themselves* and their lives. Although gender and diet-focused modules were relevant, there was uneven delivery due to the limited capacity building of nutrition volunteers. Despite these limitations, we found early marriage and dietary diversity messages nonetheless seemed to “stick.” The analysis reveals this was because these messages had simpler stories, were repeated through other modules, and had higher levels of actionability and perceived payoff.

### 5.2 Repetition and simplicity

The early marriage story was simpler and shorter than other gender-focused one. Volunteers had an easier time communicating, which seemed to result in more women being “transported” into its narrative. To exemplify this, one volunteer reported, *“the group members say that they have so many works at the home so they forget bits of stories, but I have seen that they do not forget the story of Soni [early marriage]”* (Purulia volunteer, age 29, 6/72019). Similarly, dietary diversity modules had straightforward messages – even though the issues had many complex social roots.

Early marriage and dietary diversity messages were also embedded throughout later modules, which created continuity so that women could examine interlinkages between these topics and multiple health outcomes. For example, while the protagonists of later gender-focused stories struggled with verbally abusive husbands or pressures to have large families, many women often drew connections to early marriage. One Purulia volunteer explained:

*One SHG member came and said “I am weak and not well”. I asked her when she got married and she said 13 and then had three children. I told her see this is why you are unwell - your body was not ready for this at that time.* (Purulia volunteer, age 30, 6/72019).

This quote is notable because while the story on childbirth is focused on safe delivery and birth-spacing, the respondent and nutrition-volunteer chose to focus on early marriage as the root cause of childbirth related health concerns. Thus, it seemed that for many respondents, early marriage came to denote the beginning of lives that would be filled with health or nutritional problems. Seeing early marriage as foundational to health impacted its actionability and perceived payoff, as elaborated on below.

Dietary diversity and healthy diets were the explicit focus of several modules and mentioned in most other modules. Immediately after the dietary diversity micromodule introduced the benefits from different food groups, a kitchen garden module was introduced. This addressed food access issues and reiterated dietary diversity's importance. This was followed with camps where women had their Body-Mass Index measured, which was linked to dietary intake. While most topics lasted one month, activities directly related to diet continued for 2–3 months. The repetition of messaging across other modules was evidenced in that nearly every self-help group respondent knew about dietary diversity's importance and made detailed statements on how to diversify their meals. It seemed that similar to early marriage, dietary diversity was seen as foundational for good health. One Purulia mentor summarized this consensus across sites, “*If the woman wants to be healthy and fit, the first thing she needs is nutrition, and that comes from a balanced food source”* (Purulia mentor, 6/3/2019v).

Both early marriage and dietary diversity had associated pithy phrases: ‘*kam umr ki shaadi’ (*literally “young age marriage”) and ‘*tin rang ka thali’* (literally “three-color plate,” denoting a plate with starch, vegetable, and protein). These were simple messages, and while there were complexities in putting them into practice, they were easily internalized compared to more detail-oriented “technical” messages on child feeding and disease. These modules' action points were also more tightly linked to the stories than in latter stories with multiple plot points and action messages. In particular, Ropni and Maya's stories tended to offer technical action points, whereas women connected with the stories' social aspects. This discrepancy may have presented difficulties in processing the stories into tangible actions with clear payoffs.

### 5.3 Actionability and perceived payoff

Because early marriage was implicated as a trigger of later health adversities, respondents saw stopping early marriage as critical and more manageable than using contraception (Maya) or escaping abusive home lives and excessive labor burdens (Ropni). Early marriages were also *already* seen as scandalous within villages, thus there was social desirability to avoid them. Police were increasingly enforcing early marriage laws, and there were reports of self-help groups taking collective action to stop impending early marriages. Conversely, there was no stigma or legal repercussions for women who faced verbal abuse and were overworked, or those who continued giving birth in hopes of a son. Rather, strong social norms encouraged women to be hard-working and self-sacrificing, and to bear sons. Across sites we heard similar sentiments about the challenges to enacting changes suggested in Ropni or Maya's story:

*In Bastar, there is maximum work overload and tension for the women. This [tension] is on the women only—they are stressed about income and mostly they are responsible. So after this much stress they cannot properly eat food …. These people have work overload, they do not have rest time.* (Bastar mentor, age 28, 5/29/2019)

*If somebody has a son, then it is fine [to use contraception]. But suppose somebody gives birth to two daughters. Then they are not happy and they think, ‘who would inherit the properties’? In that case, we tell them ‘what is the difference between son and daughter. Nothing.’ Then, some women argue and think about all their property.* (Purulia mentor, age 34, 6/9/2019a).

Both of these quotes illustrate how broader social norms that expected women to be selfless, hard-working, and an effective reproductive agent (that produced sons) limited the potential to enact new behaviors around women's work or reproductive health. Indeed, while confronting patriarchal norms in the context of early marriage was accepted – in part due to the legal and social repercussions - many women continued to have little power over decisions on labor allocation or family size. While these topics generated discussions, the actions they could take to address these issues were difficult to enact without family support.

The recommendation to eat diverse diets was also already viewed as socially desirable. Prior to the intervention, however, many women had no justification to prioritize dietary diversity as it seemed more of a “luxury” rather than having practical benefits. More than other messages, the outcomes linked with dietary diversity were immediate, such as improved energy and strength (to secure livelihoods) and boosted immune systems (to reduce healthcare costs). Nutrition actors in both sites related healthy diets were one of the “most important” messages because of these benefits.

*I talk most about diet and nutrition. If we eat well, we will remain healthy. Or else we will get sick. And will have to go (to the hospital).* (Bastar volunteer interview, 5/30/2019b).

*The story of three colored-food is most important, because it will increase nutrition/health. [For children] it will develop their brain. They will study more.* (Purulia volunteer FGD, 6/8/2019a).

It is noteworthy that similar to the two quotes above, most respondents linked dietary diversity to economic practicalities in their life, rather than the social or cultural benefits of consuming local nutrient-rich foods. Because the curriculum linked dietary diversity to practical economics, it had the effect of being more inclusive, where even very resource-poor women concerned with day-to-day food security could see potential benefits. There was also more flexibility in action: women were encouraged to eat a tri-color plate once daily, and if they were unable, then to try for 3–4 times per week. This flexibility contrasts other messages that consisted of binary choices, thus all women could achieve some success.

Moreover, women *enjoyed* eating diverse diets. Many fondly spoke of different local greens, lentils, or millets, and spoke authoritatively about them. Especially in Bastar, self-help group respondents were proud to learn local wild greens were especially helpful for anemia. One very shy respondent spoke excitedly when we asked her what her favorite *bhaji* (wild green) was:

*Kolyari bhaji* is very good to make with lentils. It is also good with lentils and a *Bohar bhaji,* which nowadays is available. *Torta bhaji* is also good and *moonga bhaji* is also good! (laughing …. ) We have the forest, so we get greens and vegetables without any money, and can get a complete meal. (SHG interview, 5/29/2019b)

This quote exemplifies how while there were immediate sensorial benefits of improving diets, women were emboldened knowing that it was not only pleasurable to eat well, but also practical. This seemed to go beyond economic practicality and pointed to a cultural pride rooted in local ecologies and practices that were health-sustaining. Similarly, in Purulia, where chemical agriculture was widespread, self-help group members were drawn to eating more organic vegetables from homestead land. Many discussed how community health has been negatively impacted by the use of chemical fertilizers and felt it was better to grow vegetables without using chemicals rather than spending money at the market. The modules on dietary diversity and kitchen gardens, thus, validated and encouraged women's existing practices while celebrating their cultural heritage and embodied knowledges around food.

While the *stories* discussing infant and young child feeding and neonatal health were challenging for women to recall, nearly all respondents knew the embedded *messages*, which they reported hearing from government workers. While respondents reported following these practices, mentors observed many women were unable to implement them fully due to labor burdens and poverty. Mentors in both sites observed that these stories did not resonate as much with group members, perhaps because they felt they could not act on the advice or because they had already discussed these points in government settings. In response to whether any stories did not motivate conversation in groups the mentors reported the ones about neonatal care, complementary feeding, and childhood disease were difficult because:

R1: They are about children, even after telling them they can't pay much attention to the children. They know they have to give food, but mostly they only give watery gruel. Since Maya's story is related to ourself, we tell it easily.
R-3: Yes, Maya's story (about reproductive health/family size), Ropani's story (about women's health and wellbeing) are both related to ourselves so discussing them in SHGs is not very difficult. (FGD, Bastar, 6/4/2019).

It is noteworthy that while the mentors felt Ropni and Maya's stories were easier to tell than those about child feeding, respondents reported *all stories* were difficult to actually implement due to social and economic barriers. This raises some interesting tensions in that the mentors suggest that discussing hard-to-implement messages about child health was more difficult than communicating messages about women's own health. Because women's high work burdens were central barriers to improved child feeding, perhaps women felt more compelled to critically discuss this issue *before* thinking about optimizing child feeding practices. This idea was evidenced when several mentors and volunteers in both sites noted that so long as women had high work burdens the prospects of providing more responsible childcare seemed unfeasible. One Bastar volunteer said women participants will lament, *“‘what should we do? Should we do the work or watch the children?’”* (volunteer interview, 5/30/2019a). Thus, it seemed that women implicitly understood that they could not effectively change their child feeding practices until they had more equitable labor burdens in their households. Thus, child feeding messages where women felt less power to enact immediate change were stories that may have been more difficult for them to pay attention to or critically discuss.

## 6 Discussion and conclusion

The PRADAN/PHRS nutrition curriculum was different from the behavior change communication methods used in other nutrition-sensitive agriculture programs as it addressed a wide range of messages rather than a few key action points. The intervention's curriculum was designed to utilize stories that allowed women to identify and analyze the social conditions leading to poor health, rather than using persuasive techniques of behavior change communication. It deviated from previous highly-structured participatory learning and action approaches by using a flexible format and volunteer facilitators who were not particularly accountable to deliver any set number of follow-up meetings. This format presented an opportunity to address the research questions of (i.) how volunteer-led versions of participatory learning and action might look within existing livelihood-oriented self-help groups, and (ii.) what types of messages would work best in the context of a nutrition sensitive agriculture program.

The data suggests while emotional content of stories on gender-related issues resonated strongly with women, simpler stories with one key health message (such as early marriage and dietary diversity) were more readily communicated and understood than complex stories with multiple themes. Complex stories were difficult for nutrition volunteers to communicate, and so they instead delivered key messages didactically, which women would often passively listen to or even ignore. That is, women may have identified to characters, but were not “transported” into the narrative. Volunteer facilitators had uneven literacy levels and confidence in telling the stories due to insufficient training and low accountability.

Many volunteers adept at telling stories and communicating messages were nonetheless ill-equipped to guide women through dialogical learning processes in empowering ways. Therefore, though the micromodules were *designed* with participatory learning and action tenets, due to inadequate capacity building and accountability, the curriculum was actually implemented similar to standard persuasive behavior change approaches. Resultantly, some of the more radical aims of participatory learning and action - such as mobilizing communities to demand government services - were difficult to realize. Facilitators did not always have the skill to help women contextualize their health within broader power relations, which was expressed when self-help group members felt shame around past ‘ignorance’ rather than drawing connections between marginalization and poor health. It was notable that mentors and volunteers with more substantive trainings did not express these sentiments as readily.

The factors surrounding variability in volunteer performance are beyond the scope of this analysis, but the tentative conclusions are that in order for participatory approaches to work meaningfully, proper investment in facilitators is crucial. These findings support Gugerty et al. (2018) hypothesis that well-trained facilitators are a reason why participatory learning and action interventions in self-help groups have consistently shown impact. The notion that participatory learning and action is reliant on skilled facilitators presents an interesting tension underdiscussed in the literature, whereby investing in key locals or bringing in outsiders necessarily introduces (new) power differentials that participatory learning and action approaches seek to minimize. While there is no easy solution to this tension, Narayanan and Rao (2019) instructively contend that a critical first step is to make facilitator-participant power differentials visible and use them as fodder for further critical analysis.

The wide-range of topics and use of more complex stories upon receiving community feedback was motivated by the desire to enable self-help groups to focus on issues relevant to them, and not pre-impose particular health outcomes for groups to address. Interestingly, this had the paradoxical effect of comprising the project's participatory goals, as volunteers overwhelmed with the length and number of stories resorted to communicating didactic messages. PRADAN professionals felt that information had been delivered too quickly in efforts to finish activities within the project timeline. However, because dietary diversity and early marriage themes were repeated throughout modules, women had more opportunities to interact with these messages. Tengland (2012) notes that a drawback of participatory approaches is the longer timeline it takes to achieve objectives. Similarly, Kumar et al. (2018) find that short program length is a key factor for null impact in self-help group-led nutrition projects. Without predefined objectives found in other participatory learning and action programs, participatory approaches may not be able to realistically achieve impact in 3-year project cycles. This suggests that perhaps NGOs committed to radical participation might temper expectations for impact to reflect the more deliberate processes needed for these approaches.

Critically, the messages resonating most strongly with women were about women *themselves* rather than their roles as mothers. This suggests the curriculum was filling a critical gap in health promotion within India, where women's health interventions tend to center on maternal health and infant and young child feeding. A similar program implemented in Bihar found dietary diversity to be the most relevant message in the context of self-help groups (Gupta et al., 2019). This study's findings largely align with Gupta et al. (2019), yet the qualitative data revealed that messages around women's health also invoked important discussions in self-help groups. This finding supports Ruel et al. (2018) observation that in the context of nutrition-sensitive agricultural programs, behavior change communication focused on broader objectives may be more appropriate, particularly within self-help groups.

Relatedly, we also found that women better recalled messages they had more power to take action on. Health concerns (e.g. son preference, gendered labor burdens) may have been important to women, but they expressed feeling little power to act on them. This suggests, as past studies of participatory learning have emphasized, that including men in behavior-change programming is critical. In examining a nutrition-oriented participatory project in India, Narayanan and Rao (2019) found the program's biggest successes involved men and women working together. Similarly, Rath and Nair (2010) credit the inclusion of men as critical to their participatory learning and action program's success in reducing neonatal morbidities. Thus, to engage more trenchant social determinants of malnutrition, we find it necessary to look beyond self-help group members alone.

In sum, this paper makes contributions to literature on health promotion and participatory development in two ways. First, participatory projects aimed at ethically engaging marginalized communities require intensive investment in local facilitators' skills in leading dialogical learning processes. Insufficient investments can lead facilitators to slide back into easier-to-communicate didactic messaging, which women tended to ignore. More robustly facilitated discussions may also have led women to more strongly link poor health to broader political and economic marginalization (a key goal of participatory learning and action) rather than simply their own ‘ignorance’ or local patriarchal norms. While stories engaged participants, in impoverished areas with high work burdens, stories' “transportation” function is especially critical to capture attentions. Thus, short, simple stories followed by identification, processing and discussion are key. In projects with 3–5 year timelines, difficult decisions may need to be made on prioritizing relevant issues and more slowly unpacking them to capture full benefits of dialogical learning.

Second, there is significant demand for more programs focused on women as women, rather than workers or mothers. If women's empowerment is a goal, then sufficient attention needs to be given to the physical, psychological, and emotional wellbeing of women, many of whom have faced significant trauma. Offering spaces for women to engage in dialogical learning around their bodies and wellbeing is critical. Similarly, food and nutrition offer a rich domain to honor and acknowledge women's own expertise through bringing it into conversation with established nutrition science. As food pervades women's lives in rural India, there is significant opportunity to make diverse diets a source of socio-cultural wellbeing rather than a technical subject. Women regularly prepare and consume nutritious foods that are coded as ‘poor-people food.’ Working to reverse that stigma and empower women to take pride in food heritage is a culturally compelling way to improve diets in rural India, as evidenced here.

Many health problems discussed were daunting for women to address without support. It bears reflecting on why, women alone, should be expected to enact these changes. Thus, engaging men more fully in behavior change communication programs along with continued high-level demands on states to provide quality healthcare, schools, and infrastructure are imperative for sustained reductions in malnutrition.

## Credit author statement

Carly E. Nichols was responsible for study design, data collection, data analysis, manuscript conceptualization and preparation.

## Footnotes

1 While data collection took place in a specific Block of each site, we will refer to district name to preserve respondent anonymity.

2 While PRADAN aimed to recruit only married women, in Bastar there were so few literate older women, often unmarried young women were the only literate women available.

## References

[bib1] Aboud F.E., Singla D.R. (2012). Challenges to changing health behaviours in developing countries: a critical overview. Soc. Sci. Med..

[bib2] Banks-Wallace J. (1998). Emancipatory potential of storytelling in a group. J. Nurs. Scholarsh..

[bib3] Batiwala S. (2007).

[bib5] Dutta M.J. (2007). Communicating about culture and health: theorizing culture-centered and cultural sensitivity approaches. Commun. Theor..

[bib6] FAO (2014). http://www.fao.org/3/as601e/as601e.pdf.

[bib7] Gope R.K., Tripathy P., Prasad V., Prost A. (2019). Effects of participatory learning and action with women's groups, counselling through home visits and crèches on undernutrition among children under three years in eastern India: a quasi-experimental study. BMC Publ. Health.

[bib8] Gugerty M.K., Biscaye P., Leigh-Anderson C. (2018). Delivering development? Evidence on self-help groups as development intermediaries in South Asia and Africa. Dev. Pol. Rev..

[bib9] Gupta S., Kumar N., Menon P., Pandey S., Raghunathan K. (2019).

[bib10] Hinyard L.J., Kreuter M.W. (2007). Using narrative communication as a tool for health behavior change: a conceptual, theoretical, and empirical overview. Health Educ. Behav..

[bib12] Kumar N., Scott S., Menon P., Quisumbing A. (2018). Pathways from women's group-based programs to nutrition change in South Asia: a conceptual framework and literature review. Global Food Security.

[bib13] Larkey L.K., Hecht M. (2010). A model of effects of narrative as culture-centric health promotion. J. Health Commun..

[bib14] Lentz E.C., Narayanan S., De A. (2019). Last and least: findings on intrahousehold undernutrition from participatory research in South Asia. Soc. Sci. Med..

[bib15] Malapit H. (2019). Women in agriculture and the implications for nutrition. In agriculture for improved nutrition: seizing the momentum. International Food Policy Research Institute and CABI.

[bib16] McPhail-Bell K., Bond C., Brough M., Fredericks B. (2015). “We don't tell people what to do”: ethical practice and Indigenous health promotion. Health Promot. J. Aust..

[bib17] Nair N., Tripathy P., Sachdev H.S., Prost A. (2017). Effect of participatory women's groups and counselling through home visits on children's linear growth in rural eastern India (CARING trial): a cluster-randomised controlled trial. The Lancet Global Health.

[bib18] Narayanan R., Rao N. (2019). Adult learning for nutrition security: challenging dominant values through participatory action research in Eastern India. Stud. Educ. Adults.

[bib19] Nordhagen S., Nielsen J., van Mourik T., Klemm R. (2019). Fostering CHANGE: lessons from implementing a multi-country, multi-sector nutrition-sensitive agriculture project. Eval. Progr. Plann..

[bib20] Olney D.K., Dillon A., Ruel M.T., Nielsen J. (2016). Lessons learned from the evaluation of Helen Keller International's enhanced homestead food production program. Achieving a Nutrition Revolution for Africa: The Road to Healthier Diets and Optimal Nutrition.

[bib21] Prost A., Colbourn T., Seward N., Costello A. (2013). Women's groups practising participatory learning and action to improve maternal and newborn health in low-resource settings: a systematic review and meta-analysis. Lancet.

[bib22] Rath S., Nair N., Tripathy, Prost A. (2010). Explaining the impact of a women's group led community mobilisation intervention on maternal and newborn health outcomes: the Ekjut trial process evaluation. BMC Int. Health Hum. Right.

[bib30] Rose G. (2001). Sick individuals and sick populations: 20 years later. J. Epidemiol. Commun. Health.

[bib23] Ruel M.T., Alderman H. (2013). Nutrition-sensitive interventions and programmes: how can they help to accelerate progress in improving maternal and child nutrition?. Lancet.

[bib24] Ruel M.T., Quisumbing A.R., Balagamwala M. (2018). Nutrition-sensitive agriculture: what have we learned so far?. Global Food Security.

[bib25] Sanghvi T., Seidel R., Baker J., Jimerson A. (2017). Using behavior change approaches to improve complementary feeding practices. Matern. Child Nutr..

[bib26] Tengland P.A. (2012). Behavior change or empowerment: on the ethics of health-promotion strategies. Publ. Health Ethics.

[bib27] USAID (2015). https://www.usaid.gov/global-health/health-areas/nutrition/technical-areas/effective-scale-nutrition-social-and-behavior.

[bib28] Warin M., Zivkovic T., Moore V., Jones M. (2015). Short horizons and obesity futures: disjunctures between public health interventions and everyday temporalities. Soc. Sci. Med..

[bib29] Wilson K. (2015). Towards a radical re-appropriation: gender, development and neoliberal feminism. Dev. Change.

